# Biomaterials for Corneal Regeneration

**DOI:** 10.1002/advs.202408021

**Published:** 2024-12-31

**Authors:** Yimeng Li, Zhengke Wang

**Affiliations:** ^1^ MOE Key Laboratory of Macromolecular Synthesis and Functionalization Department of Polymer Science and Engineering Zhejiang University Hangzhou Zhejiang 310058 China

**Keywords:** biomaterials, corneal regeneration, natural materials, synthetic polymers, tissue engineering

## Abstract

Corneal blindness is a significant reason for visual impairment globally. Researchers have been investigating several methods for corneal regeneration in order to cure these patients. Biomaterials are favored due to their biocompatibility and capacity to promote cell adhesion. A variety of natural and synthetic biomaterials, along with decellularized cornea, have been employed in corneal wound healing. Commonly utilized natural biomaterials encompass proteins such as collagen, gelatin, and silk fibroin (SF), as well as polysaccharides including alginate, chitosan (CS), hyaluronic acid (HA), and cellulose. Synthetic biomaterials primarily consist of polyvinyl alcohol (PVA), poly(ε‐caprolactone) (PCL), and poly (lactic‐co‐glycolic acid) (PLGA). Bio‐based materials and their composites are primarily utilized as hydrogels, films, scaffolds, patches, nanocapsules, and other formats for the treatment of blinding ocular conditions, including corneal wounds, corneal ulcers, corneal endothelium, and stromal defects. This review attempts to summarize in vitro, preclinical, and clinical trial studies relevant to corneal regeneration using biomaterials within the last five years, and expect that these experiences and outcomes will inspire and provide practical strategies for the future development of biomaterials for corneal regeneration. Furthermore, potential improvements and difficulties for these biomaterials are discussed.

## Graphical abstract

A variety of natural and synthetic biomaterials, along with decellularized cornea, have been employed in corneal wound healing. The current progress of biomaterials is summarized for corneal regeneration, and expect that these experiences and outcomes will inspire and provide practical strategies for the future development of corneal regeneration. Furthermore, potential improvements and difficulties for these biomaterials are discussed.

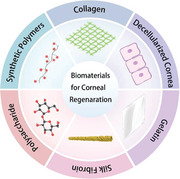

## 1 Introduction

Light enters the eye through the cornea, a transparent and multi‐layered tissue that guides it to the retina via the lens. The cornea is made up of three cellular regions: epithelium, stroma, and endothelium, that are divided by two non‐cellular layers named Bowman and Descemet membranes.^[^ ^1^, ^2^ ^]^ About 57 million people throughout the world are blind due to corneal diseases or accidents, such conditions include bullous keratopathy, keratoconus, and scarring that causes irreversible loss of clarity.^[^ ^3^ ^]^ While corneal blindness can be treated through transplantation, an estimated 12.7 million people are currently awaiting a donor cornea, with only one cornea available for every 70 required.^[^ ^4^, ^5^ ^]^

To overcome barriers to the corneal supply shortfall, biomaterials have attracted the curiosity of researchers as an alternative therapy option for eye illnesses. The corneal regeneration biomaterials should be biocompatible, transparent, strong, and moisture‐resistant.^[^ ^6^ ^]^ Additionally, corneal biomaterial constructs have the ability to replicate the microenvironment of the original tissue that stimulates cell migration, adhesion, differentiation, and proliferation. With the advancement of regenerative medicine, a wide range of biomaterials have been developed for corneal repair.^[^ ^7^ ^]^

**Figure 1:**
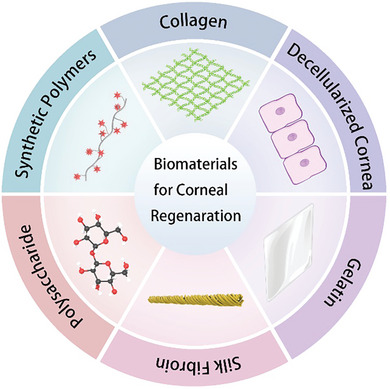
Biomaterials for corneal regeneration.

Bioengineered stromal scaffolds, tissue adhesives, 3D bioprinting, exosomes, stem cells, gene therapy, nanomedicine, trophic factors, and small molecule drugs are the methods reviewed in previous works that aim to restore the transparency and function of the cornea.^[^ ^6^, ^8^, ^9^, ^10^, ^11^, ^12^, ^13^ ^]^ Researchers have reviewed specific material preparation techniques^[^ ^1^, ^11^, ^14^, ^15^ ^]^ and applications of natural biomaterials.^[^ ^7^, ^16^ ^]^ Besides, Girolamo reviewed several biological scaffolds as carriers for stem cells in the treatment of limbal stem cell deficiency recently.^[^ ^17^ ^]^ Having said that no review devoted to biomaterials for corneal regeneration caused by multiple diseases or injuries has been located. The objective of this research is to offer a thorough and concise overview of the studies mainly in the last five years about biomaterials including collagen, decellularized cornea, gelatin, silk fibroin, and polysaccharides in addition to several synthetic polymers for corneal regeneration (**Figure** **1**, **Table** **1**).

## 2 Collagen

Seventy percent by weight of the cornea is composed of collagen, the principal component of the stromal layer.^[^ ^18^ ^]^ While other types of collagens are present in lesser quantities, the most prevalent protein in the cornea is type I collagen (Col‐I).^[^ ^6^ ^]^ Collagen has thus been a widely utilized material in the construction of scaffolds that emulate the composition of the indigenous cornea.^[^ ^19^ ^]^

**Figure 2:**
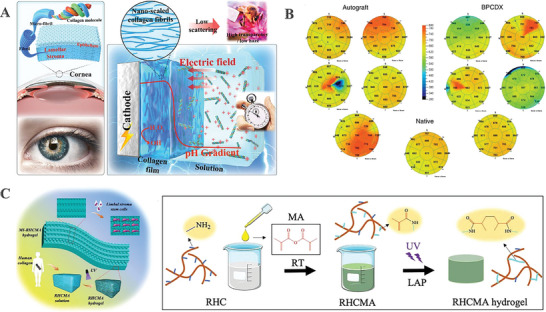
Development and evaluation of collagen‐based corneal substitutes. A) Schematic showing the electrochemically triggered kinetic assembly of collagen I. Adapted with permission.^[^ ^24^ ^]^ Copyright 2022, American Chemical Society. B) Six months after surgery, pachymetry maps depicting corneal thickness with thickness color‐coded. Adapted with permission.^[^ ^25^ ^]^ Copyright 2022, The Authors. C) Recombinant human collagen hydrogel featuring microstructures arranged in a hierarchical fashion. Adapted with permission.^[^ ^26^ ^]^ Copyright 2024, Elsevier B.V.

Collagen fibrils, which have a tiny diameter of 25–35 nm, are densely packed and placed uniformly across the stromal layer to create orthogonal layers or lamellae. Each lamella consists of ≈200 collagen fibrils that are aligned parallel to one another and perpendicular to the neighboring lamellae.^[^ ^20^, ^21^ ^]^ The exact spatial arrangement of collagen fibrils is the basis of its mechanical strength and transparency. The notion given by Maurice states that the transparency is caused by the lattice configurations of the fibrillar collagen in the stroma.^[^ ^22^ ^]^ Chen et al., used pure electro‐compacted collagen (EC) to fabricate a 3D corneal stroma model with orthogonal orientation. EC films are composed of collagen fibrils that are aligned and provide support for primary human corneal stromal cells. Films formed more quickly with the optimized current density, and there were no bubbles, which would other cause poor mechanical characteristics and low transparency.^[^ ^23^ ^]^ Another study further demonstrated the production of an artificial corneal replacement made of collagen, which had superior optics due to its structure being defined and controlled at the nanoscale using an electro‐assembly technique (**Figure** **2**A). Specifically, the cathode electrode reaction induced triple helix collagen molecules to assemble into microfibers at their isoelectric point (Ip = 4.5). Then these microfibrils were integrated into collagen hydrogel film under intermolecular interactions such as hydrogen bonds. Structural/optical characterization showed that this fine, uniform structure enabled collagen to have low haze and high transparency.^[^ ^24^ ^]^

As the primary constituent of the endogenous corneal stroma, collagen demonstrates remarkable biocompatibility and thus emerges as an excellent scaffold material for corneal regeneration. Nevertheless, animal‐derived collagen exhibits certain limitations, including inadequate light transmission and vulnerability to enzymatic degradation. As a consequence, crosslinking collagen is an essential process for enhancing its physicochemical characteristics.^[^ ^27^ ^]^ Riboflavin‐A (UV‐A) light curing and polyethylene glycol are recent technologies utilized for crosslinking, showing good biocompatibility. The crosslinked gel can create an adhesive connection with nearby collagen.^[^ ^28^, ^29^ ^]^ In addition, the utilization of 1‐ethyl‐3‐(3‐dimethylaminopropyl) carbodiimide (EDC) and N‐Hydroxy succinimide (NHS) in the production of crosslinked collagen membranes enhances both optical and mechanical characteristics, but it should be noted that the durability of implants stabilized by EDC/NHS is restricted over extended periods of time. Wang et al., chose EDC/NHS as the second cross‐linking ingredient to construct a double cross‐linked membrane to achieve greater suture strength and stability, based on pure collagen being conjugated with polyrotaxane multiple aldehydes (PRAs).^[^ ^27^ ^]^ Rare earth metal nanoparticles have greater cross‐linking effectiveness compared to standard collagen crosslinking agents. Additionally, several of them have anti‐inflammatory and anti‐oxidative properties. Polysaccharides can cause glycosylation of collagen, leading to collagen cross‐linking. Vijayan et al., developed a bio‐composite hydrogel with collagen, dextran, and gadolinium oxide nanoparticles for corneal tissue regeneration.^[^ ^30^ ^]^ Simultaneously, the creation of corneal stroma‐mimicking biomaterials using vitrification, a controlled dehydration method, led to a progressive rise in collagen concentration and organization. Crucial for in vivo implantation using simple interrupted sutures, this innovative approach allowed the collagen to possess great optical transparency and strong mechanical integrity.^[^ ^31^, ^32^ ^]^

The process of corneal matrix healing is an intricate physiological mechanism that impacts the development of corneal scars, so it is also one of the important factors affecting corneal transparency. On the basis of finding materials for repairment, it is an issue that requires resolution to inhibit scar formation during corneal healing. MicroRNAs are small RNA molecules that control gene expression. Specifically, microRNA‐133b (miR‐133b) can influence the levels of alpha‐smooth muscle actin (α‐SMA) and Col‐I in corneal stromal cells by binding to target mRNA and preventing the translation of α‐SMA and Col‐I. Zhao et al., utilized nanocomplexes consisting of gold nanoparticles (AuNPs) and miR‐133b within the collagen‐based substance, aiming to facilitate corneal regeneration and prohibit scarring. Results of lamellar keratoplasty in vivo demonstrated that the cornea epithelized fully and quickly, and corneal transparency could persist after transplantation.^[^ ^33^ ^]^ Mesenchymal stem cells (MSCs) and their secreted factors have been researched because of their ability to reduce inflammation and inhibit blood vessel formation. Using multi‐functional polyethylene glycol (PEG)‐succinimidyl esters to cross‐link collagen gels and encapsulate MSCs is a viable method for delivering the secretome of immobilized MSCs. The gel is efficient in treating ocular inflammatory conditions, such as alkali burns.^[^ ^34^ ^]^

Recombinant human collagen (RHC) exhibits reduced heterogeneity and poses a minimal risk of zoonotic disease transmission in comparison to the majority of commercially available collagen, derived from animal tissues.^[^ ^35^ ^]^ Plant‐derived recombinant human collagen type I (RHCI) was crosslinked with EDC/NHS to form hydrogels. In contrast to yeast‐produced collagen and corneal allograft transplantation, the primary benefit of this strategy was its extraordinarily high yield.^[^ ^36^ ^]^ After the modification of the RHC with methacrylate anhydride (RHCMA), Kong et al., fabricated a hierarchically ordered hydrogel with aligned microgrooves and a periodically arranged nano‐scale porous structure (MI‐RHCMA) by combining the lithography and photonic crystal template techniques (Figure 2C). Animal experiments involving male SD mice demonstrated that the RHCMA hydrogel possessed a microstructure organized hierarchically, and could expedite the process of tissue repair and ultimately permit in vivo regeneration of the damaged stroma tissue.^[^ ^26^ ^]^

Making recombinant human collagen in large quantities is challenging due to the fact that it requires complex post‐translational modifications and the ordered structure of collagen to self‐assemble. A bioengineered pig collagen (BPC) platform was suggested by researchers. It is made from high‐purity medical‐grade collagen derived from swine skin and designed to endure surgical implantation in the eye. The bioengineered porcine construct (BPCDX), a transparent implantable hydrogel, was created through the application of dual chemical and photochemical crosslinking. Researchers inserted BPCDX into 20 individuals with advanced keratoconus to alter the original corneal stroma. No adverse events were recorded during the 24‐month follow‐up period. Improvements in corneal thickness were documented with an average rise of 209 ± 18 µm in India and 285 ± 99 µm in Iran (Figure 2B).^[^ ^25^ ^]^ A cell‐free, liquid hydrogel matrix called LiQD cornea is made up of fibrinogen combined with polyethylene glycol and short collagen‐like peptides. It is an example of a fully specified, synthetic collagen analog that is self‐assembled and far less expensive than RHC. The composition comprised abbreviated collagen‐like peptides (CLP) connected with PEG and mixed with fibrinogen or 2‐methacryloyloxyethyl phosphorylcholine (MPC), crosslinked using 4‐(4,6‐dimethoxy‐1,3,5‐triazin‐2‐yl)‐4‐methylmorpholinium chloride (DMTMM).^[^ ^37^, ^38^ ^]^ This hydrogel filler's biocompatibility and biofunctionality enabled the gradual rebuilding of corneal transparency and structure in an animal model, providing a viable substitute for corneal transplantation and cyanoacrylate glue in the treatment of ulcerated corneas.^[^ ^39^ ^]^

Fish scales undergoing decellularization, decalcification, and bending could be utilized in the fabrication of fish‐scale collagen membranes (FSCM). This membrane exhibited remarkable transparency, sufficient water content, and favorable biocompatibility. In vivo investigation demonstrated that the cultivated corneal endothelial cells (CECs) affixed to the FSCM resembled healthy CECs in terms of viability and morphology.^[^ ^40^ ^]^

Additionally, the amniotic membrane (AM), the innermost layer of the placenta, has been demonstrated to be a reliable and stable matrix for corneal regeneration.^[^ ^41^ ^]^ In most tissue engineering applications, the concept of AM typically denotes the basement membrane,^[^ ^42^ ^]^ which mainly comprises collagens types III, IV, and V.^[^ ^43^ ^]^ Owing to its preferable biocompatibility, AM can serve as a carrier for human keratinocytes and limbal stem cells, and markedly decrease the culture time necessary for preparing cell cultures for clinical application in patients with ocular chemical burns.^[^ ^44^ ^]^ Zhao et al., additionally proposed the supplementation and sustained release of growth factors in AM through the surface grafting of heparin (AM‐HEP) to enhance corneal epithelial wound healing by the supplement and sustained release of epithelial growth factor (EGF).^[^ ^45^ ^]^ To enhance the inadequate mechanical strength and elevated biodegradation rate of AM, researchers have suggested several effective laboratory solutions, including the electrospinning of polydimethylsiloxane (PDMS) onto the surface of decellularized AM (AM/PDMS),^[^ ^46^ ^]^ the fabrication of a composite decellularized AM (dAM) membrane utilizing bioabsorbable poly(ε‐caprolactone) (PCL) nanofiber mesh,^[^ ^47^ ^]^ and the integration of decellularized AM with elements of natural Descemet membrane (mcdAM).^[^ ^48^ ^]^

These in vitro and in vivo experiments indicate that collagen‐based biomaterials, with straightforward modifications, show potential for ophthalmic applications, especially in corneal tissue engineering and regeneration. In order to put it into clinical application, the cost of mass production should also be considered.^[^ ^49^ ^]^

## 3 Decellularized Cornea

The extracellular matrix (ECM) contains numerous biomolecules including structural proteins, glycosaminoglycans (GAGs), and tiny molecules that are essential for tissue maintenance and healing.^[^ ^50^ ^]^ In organ decellularization, cells, and their detritus are eliminated to produce an acellular scaffold made up solely of the organ ECM. Major histocompatibility complexes are depleted through cell removal, thereby decreasing the likelihood of graft rejection.^[^ ^51^ ^]^ Although cells and immunogenic molecules are largely eliminated, biomaterials derived from the decellularized extracellular matrix (dECM) preserve the complex collagen architecture of corneal stroma, which is challenging to reproduce with alternative biomaterials.^[^ ^52^ ^]^

Decellularization cornea has been the subject of investigation through various methodologies, that can be broadly classified into three categories: physical, chemical, and biological.^[^ ^53^ ^]^ Freeze‐thawing cycles are the most often used physical approach.^[^ ^54^, ^55^ ^]^ Chemical agents may include ionic detergents,^[^ ^56^, ^57^ ^]^ non‐ionic detergents,^[^ ^58^ ^]^ hypertonic or hypotonic salt solutions,^[^ ^59^ ^]^ and acids or bases.^[^ ^60^ ^]^ Enzymes have been utilized as biological techniques for decellularization.^[^ ^61^ ^]^

**Figure 3:**
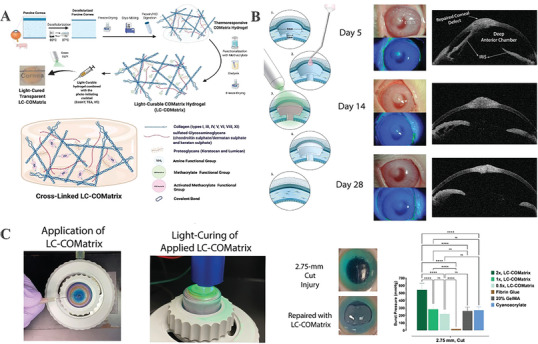
Development and evaluation of dECM‐based corneal substitutes. A) The fabrication process of LC‐COMatrix hydrogel.^[^ ^54^ ^]^ B) Rabbit corneal macroperforation healed in vivo using LC‐COMatrix hydrogel.^[^ ^54^ ^]^ C) In vitro repairs of corneal penetrations and perforations were used to evaluate the attachment strength of the LC‐COMatrix hydrogel. Burst pressure measurements were taken on a 2.75 mm full‐thickness cut injury. Adapted with permission.^[^ ^54^ ^]^ Copyright 2022, Wiley‐VCH GmbH.

Autologous and allogeneic tissue sources are very rare. Xenogeneic acellular extracellular matrix is a prominent focus in current research on tissue restoration technologies. Pigs are a significant source of diverse extracellular matrix.^[^ ^62^ ^]^ Out of all the species studied, porcine corneal matrix protein is the most similar to human corneal matrix protein.^[^ ^54^ ^]^ Yazdanpanah et al., described a thermoresponsive hydrogel using decellularized porcine cornea matrix (COMatrix) that transformed from a liquid into a gel at 37 °C. The COMatrix, consisting of different collagens, sulfated glycosaminoglycans, and proteoglycans such as lumican, keratocan, and laminin, could serve as a bandage for the ocular surface to improve the recovery of corneal epithelial wounds. Nevertheless, the biomechanical properties and stability required for the restoration or substitution of corneal stromal deficiencies had been eliminated from the COMatrix.^[^ ^60^ ^]^ Consequently, researchers attempted to refine and establish a procedure for functionalizing the COMatrix hydrogel onto a light‐curable cornea matrix (LC‐COMatrix). LC‐COMatrix achieved a biomechanical strength like that of the human cornea as preserving its optical and biological characteristics after light curing (**Figure** **3**A). In this work, the adhesiveness characteristics for LC‐COMatrix (4 min light curing) were reported to be 542 ± 86 mmHg, notably surpassing the burst pressure of fibrin glue or cyanoacrylate (Figure 3C). In rabbit models, the LC‐COMatrix successfully closed substantial corneal holes, filled partial corneal stromal defects, and seamlessly merged with the surrounding tissue. Still, its capacity to fill a full‐thickness corneal incision was not entirely feasible (Figure 3B).^[^ ^54^ ^]^ Likewise, corneas donated from bovine eyes (dCMH) can be utilized to create thermosensitive hydrogels following decellularization.^[^ ^59^ ^]^

Corneal collagen fibers exhibit a high level of organization in hexagonal space lattice structures due to the hydrogen bond interactions of GAGs. The natural hydration level of the cornea is changed during decellularization as GAGs attached to collagen fibers are impaired, causing them to lose their electrostatic force. As a result, this can lead the host corneal stroma cells to regulate the recovery of corneal transparency to take longer.^[^ ^6^ ^]^ The acellular porcine corneal stroma (APCS) was prepared using phospholipase A2 decellularization and subsequently crosslinked with aspartic acid (Asp). Hydration and ultrastructure regularity were significantly higher in the modified APCS‐Asp scaffold compared to APCS. It was able to restore the original structure and visual capabilities of the cornea after a week and demonstrated good results in the later stages of tissue and nerve regeneration.^[^ ^61^ ^]^

Ocular sealants have emerged as a viable technique for treating corneal stromal damage. Given that cornea‐derived dECMs (Co‐dECM) in hydrogel form include numerous tyrosine‐carrying proteins, Kim et al., created Co‐dECM‐based sticky sealants that can be used in ocular tissue reconstruction for deep tissue wounds.^[^ ^58^ ^]^ The functional branches were distributed throughout the dECM hydrogel to facilitate rapid crosslinking and further used a ruthenium/sodium persulfate (Ru/SPS) system to synthesize catecholic amino acid of L‐3, 4‐dihydroxyphenylalanine (Dopa), whereby the conversion of tyrosine to Dopa promoted adhesiveness.^[^ ^63^ ^]^ Four weeks after the application of the gelatinized Co‐dECM (GelCodE) sealant onto a rabbit model of a corneal defect, the thickness of the cornea (332.3 ± 10 µm) matched that of a typical cornea (346.1 ± 21 µm). As determined by Image J software (NIH), the corneal opacity of the GelCodE sealant groups was comparable to that of a healthy cornea. Accordingly, the GelCodE sealant facilitated the restoration of corneal transparency and scarless tissue with in vivo compatibility. Furthermore, Fibrin‐dECM, a human fibrin sealant containing Co‐dECM microparticles, was described by Chandru et al., as a potential alternative to traditional corneal anterior stromal reconstruction techniques.^[^ ^64^ ^]^

Bioprinted scaffolds are also among the most common biomaterials containing dECM particles. Drops of dECM bioink can be carefully placed in a pattern that matches the contours of a damaged organ or tissue. It is typically made by solubilizing pepsin. Bioprinted scaffolds may recreate the diverse features of multilayer ECM structures by utilizing extrusion‐based and laser‐assisted bioprinters for design and patterning.^[^ ^52^ ^]^ The Co‐dECM bio‐ink can 3D print with enclosed cells and has high transparency because of its intricate fibril structures. The specific alignment of collagen fibrils with the correct diameter and spacing allows visible light to pass through the cornea. The thickness and curvature of an artificial cornea can be modified and fabricated to suit the needs of a specific patient.^[^ ^65^ ^]^ We anticipate that 3D‐printed corneas will yield numerous advantages, including great adaptability, consistency, and repeatability for translational research.

In summary, the dECM matrix can guarantee the preservation of native structures and significant compositions, creating a microenvironment that facilitates tissue repair, regeneration, and restoration. Nevertheless, it is important to note that the decellularization process has the potential to cause certain damage to the original structure and properties of ECM, and also induce immunogenicity with remaining cellular components.^[^ ^66^, ^67^ ^]^ That is why it is crucial to optimize decellularization protocols in order to mitigate toxicity.

## 4 Gelatin

Gelatin is a natural protein generated from collagen hydrolysis. It can be divided into two types: acid‐hydrolyzed gelatin and base‐hydrolyzed gelatin depending on the acid or alkaline/base pre‐treatment and heat denaturation of collagen.^[^ ^68^ ^]^ Gelatin is a biocompatible, inexpensive, and low‐immunogenic material with numerous potential applications in tissue engineering.^[^ ^69^ ^]^ For endothelial cell transplantation, gelatin showed superior mechanical qualities, permeability, and transparency when compared to atelocollagen sheets.

Gelatin is inherently thermally unstable and undergoes rapid degradation unless chemically crosslinked or combined with other materials.^[^ ^6^ ^]^ Earlier studies showed that the o‐nitrosobenzaldehyde group (NB) had a robust adhesive capacity to amino groups in tissue.^[^ ^70^ ^]^ Zhang et al., created a hydrogel coating technique using gelatin that has been altered to include NB. GelNB, a collagen derivative modified by NB, may be able to withstand quick removal and offer an ideal environment for the spread and restoration of corneal cells. Moreover, once the photo‐activated GelNB solution reached the eyes, the hydrogel coating would develop on the corneal defect.^[^ ^71^ ^]^ Romo–Valera et al., described a technique for cross‐linking gelatin with lactose via the Maillard reaction, a non‐enzymatic glycation, or with citric acid, a tricarboxylic acid that created amide bonds by using a nucleophilic reaction between the amino groups and the carboxyl groups.^[^ ^72^ ^]^

**Figure 4:**
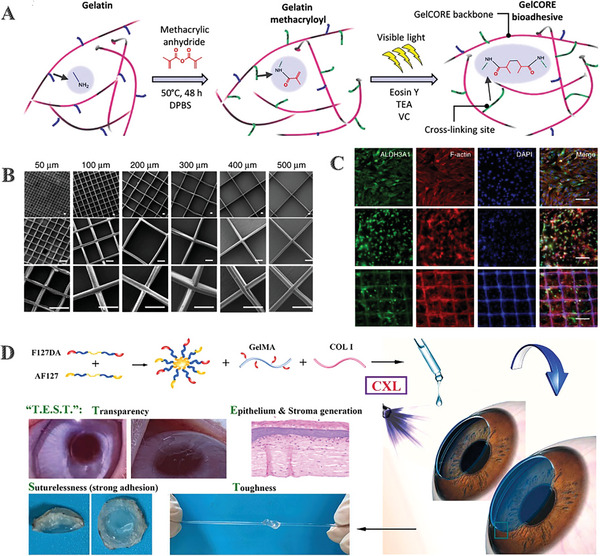
Development and evaluation of gelatin‐based corneal substitutes. A) Chemical reaction schematic for the formation of GelCORE and the photocrosslinking of the prepolymer solution. Adapted with permission.^[^ ^77^ ^]^ Copyright 2019, The Authors. B) SEM captured at different magnifications of the grid scaffold demonstrates fiber spacing varying between 50 and 500 µm.^[^ ^78^ ^]^ C) Following 7 days of culture, LSSCs in SF medium on 2D TCPs, 3D GelMA, and the 100G construct were stained for cytoskeleton and ALDH3A1 expression. Adapted with permission.^[^ ^78^ ^]^ Copyright 2022, The Authors. D) Formation, application, and graphical representation of a light‐curable adhesive corneal cross‐linking hydrogel patch for the cornea. Adapted with permission.^[^ ^79^ ^]^ Copyright 2023, The Authors.

In a different approach, methacrylic anhydride can be employed to chemically modify gelatin, resulting in the formation of GelMA, a substance adaptable to crosslinking when exposed to UVA radiation. Because of its matrix metalloproteinase‐degradable amino acid chain and cell‐adhesive RGD (arginine, glycine, and aspartate) patterns, GelMA can promote the adhesion, dissemination, and proliferation of numerous cell types.^[^ ^73^ ^]^ According to the findings of Yan et al., rat bone marrow MSCs (rBM‐MSCs) grew and proliferated more positively in 7 wt.% GelMA hydrogel. Conversely, the 30 wt.%GelMA hydrogel exhibited superior mechanical properties and may have been more conducive to promoting rBM‐MSC differentiation into keratocyte‐like cells.^[^ ^74^ ^]^ With a GelMA‐based bio‐ink and a visible light Stereolithography (SLA) 3D bioprinting system, Mahdavi et al., bio‐printed the precise geometry of the human corneal stroma in preparation for tissue regeneration, and GelMA was photo crosslinked using a visible light‐based photoinitiation system that included Eosin Y, triethanolamine (TEA), and 1‐vinyl‐2‐pyrrolidinone (NVP). The scaffold was constructed using 12.5 wt.% GelMA demonstrated exceptional compatibility with corneal stromal cells.^[^ ^75^ ^]^ Microscale hydrogel scaffolds were created by bioprinting with digital light processing (DLP) and either hyaluronic acid glycidyl methacrylate (HAGM) or GelMA. The limbal stem/progenitor cells (LSCs) were shown to be active in GelMA‐based scaffolds but inactive in HAGM‐based scaffolds. Zhong et al., created a new bio‐printed dual ECM “Yin‐Yang” model containing LSCs to maintain both active and passive states based on these findings.^[^ ^76^ ^]^ Sani et al., developed GelCORE, a gelatin‐based adhesive biomaterial that can be utilized to repair corneal stromal defects quickly and permanently (**Figure** **4**A). Because of its regenerative and adhesive characteristics, the material can accurately simulate the natural process of corneal tissue healing.^[^ ^77^ ^]^

**Figure 5:**
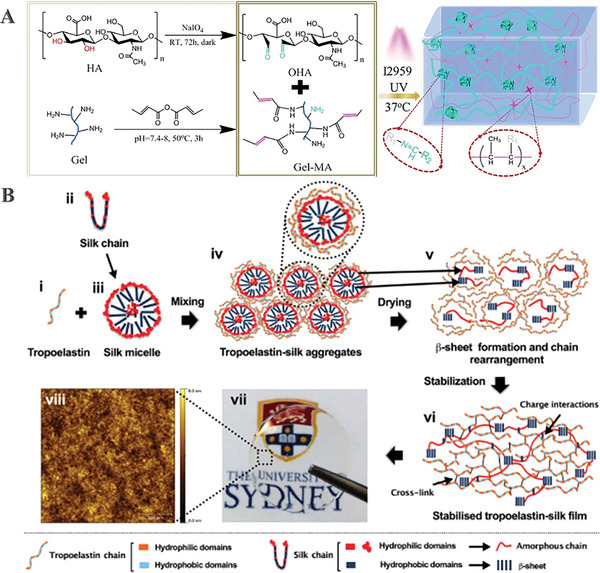
Development of gelatin‐based and SF‐based corneal substitutes. A) Schematic diagram of the GelMA/OHA hydrogel formation. Adapted with permission.^[^ ^81^ ^]^ Copyright 2022, Elsevier B.V. B) Proposed model of interaction between tropoe‐lastin and silk with its photographic image and Atomic Force Microscopy image. Adapted with permission.^[^ ^83^ ^]^ Copyright 2020, Elsevier B.V.

As a way to optimize its mechanical characteristics, cell flexibility, and rate of decay, gelatin has been in combination with hyaluronic acid, collagen, and synthetic polymer. Kong et al., accurately created orthogonally aligned sub‐micron fibers made of poly (ε‐caprolactone)‐poly (ethylene glycol) (PECL) by putting GelMA fluid into a mold with a grid scaffold (Figure 4B). Grid fibers have the potential to greatly enhance GelMA's compressive modulus. The placement of microfibers within the GelMA hydrogel affected cell organization and morphology in the 100G (using the mold with the 100 µm grid scaffold) structure. In addition, the serum‐free SF media were supplemented with insulin, β‐FGF, and ascorbic acid. The results demonstrated that these factors upregulated keratocyte‐specific gene and protein expression (ALDH, KERA, AQP1) while simultaneously preserving keratocyte‐like traits(Figure 4C).^[^ ^78^ ^]^ Rose et al., investigated electrospun gelatin and PCL scaffolds as biomaterials for ocular stromal tissue regeneration.^[^ ^80^ ^]^ To enhance interface adhesion and cohesion, Li et al., incorporated Col‐I into GelMA‐based hydrogel, as well as assure transparency and biocompatibility. Additionally, they co‐assembled nano‐micelles of Pluronic F127 diacrylate (F127DA) and Aldehyded Pluronic F127 (AF127) into GelMA. The co‐assembled micelle presence greatly limited the hydrogel expansion and imparted hardness to the hydrogel. The aldehyde groups within the micelle, the link between Col‐I and the ocular ECM caused by CXL, as well as the light‐curable networks produced in situ collaborated to give the hydrogel outstanding tissue adhesive properties (Figure 4D).^[^ ^79^ ^]^ Wang et al., developed a double‐network hydrogel by combining GelMA and hyaluronic acid, which were oxidized using sodium periodate to produce an aldehyde group known as oxidized hyaluronic acid (OHA) (**Figure** **5**A). The hydrogel precursors could speed up epithelial formation and stimulate the regeneration of a corneal equivalent with a structure resembling that of the natural cornea when injected into the specific location.^[^ ^81^ ^]^ Additionally, Nguyen et al., used gelatin microcarriers to repair corneal damage by taking advantage of the effects of aldehyde OHA's oxidation level. High oxidation levels, in particular, could provide the microcarrier surface with a smooth topography, strong stiffness, and a substantial quantity of OHA grafting. In a rabbit model of corneal alkali burn injury, injecting keratocytes/functionalized microcarriers with a suitable oxidation level directly into the cornea significantly reduced corneal swelling by ≈62 times, restored collagen production and keratocan expression to ≈94% and 89% respectively, and restored the disordered collagenous stromal tissue within 4 weeks.^[^ ^82^ ^]^

Gelatin's isoelectric point enables the formation of a polyion complex, making it suitable as a carrier for growth factors and biomolecules. Hydrogels made from GelMA derived from codfish skin were created using photocrosslinking in a study and then filled with ascorbic acid (AA).^[^ ^84^ ^]^ Farasatkia et al., created an effective double‐layer film including an ascorbic acid reservoir sodium alginate (SA) adhesive and an anisotropic layer consisting of micro‐patterned silk nanofibrils (SNF) with GelMA. The film can strongly adhere to the epithelium and steadily supply ascorbic acid. Ascorbic acid increased the cells' biological activity, aiding in the regeneration of the corneal stroma.^[^ ^85^ ^]^

Gelatin's low cost, minimal antigenicity, and excellent biocompatibility render it suitable for corneal regeneration. Still, its high biodegradability and poor mechanical properties restrict its practicality, necessitating the development of composites or chemical modifications. To develop a novel polymer for a gelatin‐based material, the concentration of the products, reaction duration, reaction temperature, and drying temperature are critical to prevent denaturation of the gelatin proteins. The widespread use of gelatin‐based hydrogels may be constrained by their relatively intricate preparation methods.

## 5 Silk Fibroin

Silk fibroin (SF) is a natural biomaterial derived from silkworms and can be refined by alkali or enzyme‐based degumming processes to remove the sericin protein. Fibroin is utilized independently or in conjunction with other compounds in various forms, including hydrogels, films, or porous scaffolds.^[^ ^86^ ^]^

SF from Bombyx mori cocoons (BM) has been widely studied for cornea engineering. Still, Ramachandran et al., found that silk protein from non‐mulberry varieties Philosamia ricini (PR) and Antheraea assamensis (AA) showed superior support for corneal epithelial (CE) cells compared to BM.^[^ ^87^ ^]^

SF films produced by a basic solvent casting method are permeable, strong, thick, and transparent, so they're adopted to convey corneal epithelium and limbal cells. Research has combined SF with other chemicals or materials to produce films that can be used to create endothelium grafts. Song et al., introduced a transparent and stable film made from glycerol‐modified silk fibroin (G/SF); in comparison to the SF film, it had a thinner profile, a coarser surface, and a more uniform structure. In vitro assays indicated that G/SF films could slightly increase the initial adhesion and proliferation rate of corneal endothelial cells (CEnCs) when contrasted with SF films.^[^ ^88^ ^]^ By mixing mechanically strong and long‐lasting protein silk with physiologically active tropoelastin, Aghaei‐Ghareh–Bolagh et al., created tropoelastin‐silk hybrid films. Silk islands, which acted as cross‐linking sites, electrostatic contacts, and hydrophobic connections were all components of these films, which were dense networks of tropoelastin molecules (Figure 5B). Physically, the film closely resembled the natural cornea and provided support for the growth and function of both corneal epithelial and endothelial cells.^[^ ^83^ ^]^

SF product properties are typically improved through the implementation of postfabrication procedures. Sun et al., showed that a broad range of silk fibroin film stiffness may be controlled by basic methanol processing, which in turn influenced cell spreading and actin cytoskeletal tension.^[^ ^89^ ^]^

However, there are some limitations to consider. SF has a poor degradation rate and a yellowish hue. One way to speed up the decomposition rate of SF is by combining it with fast‐degrading compounds like gelatin. The silk fibroin/gelatin (SF/G) corneal film underwent significant enzymatic degradation in vitro, with over 90% destroyed within 48 h.^[^ ^90^ ^]^ Recently, a hybrid scaffold made of PCL‐SF was created using an electrospinning method. Different weight ratios of aligned and non‐aligned PCL‐SF scaffolds were manufactured. Compared to other scaffolds, the orientated PCL‐SF (60:40 and 50:50) ones were particularly transparent, hydrophilic, water‐absorbing, and in vitro‐breakdown‐rate‐friendly.^[^ ^91^ ^]^ Gavrilova et al., demonstrated that biodegradable SF scaffolds containing glial cell line‐derived neurotrophic factor (GDNF) facilitated the repair of the cornea's epithelial‐stromal defect and stimulated the regrowth of corneal nerves following injury.^[^ ^92^ ^]^

SF and other mucoadhesive polymers are frequently used to increase the effectiveness of eye medicines. Barroso et al., rapidly created gentamicin‐loaded methacrylated‐silk (SilkMA) hydrogels by methacrylating silk and exposing it to low‐intensity UV light. They verified that the material's biocompatibility and in vitro degradation were unaffected by methacrylation through examining fibroblast adhesion and proliferation. Agar diffusion experiments indicated that the produced hydrogels effectively prevented the growth of Staphylococcus aureus and Pseudomonas aeruginosa for 72 h.^[^ ^93^ ^]^ Bhattacharjee et al., explained the process of creating semi‐interpenetrating hydrogel by physically trapping SF within a 3D hydrophilic network made of polyacrylamide (PAM). In this case, SF could be essentially trapped in the empty region of the highly cross‐linked PAM network, and there would be no requirement for a chemical crosslinker. As shown in the results, SF at levels of 6 and 8 wt.% facilitated cell adhesion while maintaining optimal thermal stability and adsorption performance, both of which are beneficial for corneal regeneration therapeutics. This research serves as a preliminary step in the development of clinically applicable ocular implants by fabricating semi‐interpenetrating network hydrogels based on SF.^[^ ^94^ ^]^

Generally, scientists extensively utilize SF in corneal regeneration owing to its biocompatibility, biodegradability, mechanical strength, and structural tunability. Even so, its vulnerability to yellowing has not been adequately addressed. Furthermore, the seasonality and provenance of mulberry leaves may influence the batch characteristics of SF.^[^ ^95^ ^]^

## 6 Polysaccharide

Biomaterials derived from polysaccharides have been intensively researched as scaffolding systems in cornea tissue engineering. GAGs are prevalent in the extracellular matrix of corneal tissue and are linear polysaccharides. Consequently, cornea tissue engineering can partially mimic the biological cues of this tissue through the use of polysaccharides.^[^ ^50^ ^]^ In the field of corneal tissue engineering, polysaccharides are favorable due to their exceptional biocompatibility, flexibility, processability, and affordability.^[^ ^96^ ^]^

### 6.1 Alginate

Alginate, a ubiquitous natural heteropolysaccharide, is found in a wide variety of brown algae species. The ratio of b‐d‐mannuronic and a‐l‐guluronic acid chains, which affect the material's physical properties, determines the composition.^[^ ^97^ ^]^

**Figure 6:**
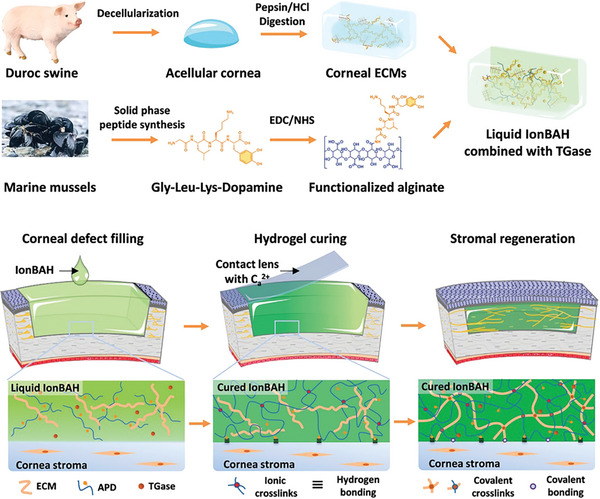
Schematic illustration of IonBAH. Adapted with permission.^[^ ^99^ ^]^ Copyright 2022, Wiley‐VCH GmbH.

Alginate and its composites are significant biomaterials due to their various biological applications. Hydrogels based on alginate have been studied for corneal healing therapies in preclinical and clinical settings.^[^ ^7^ ^]^ Without the need for additional chemical crosslinking agents, an in situ hydrogel was produced by the aldehyde groups of sodium dodecylbenzylamide (SAD) self‐crosslinking with free amino groups of carboxymethyl chitosan (CMCTS). This hydrogel was then successfully used to transplant encapsulated LSCs to alkali burned corneas in a rabbit model, resulting in quick tissue regeneration and wound healing.^[^ ^98^ ^]^ A dual‐network hydrogel consisting of peptide‐modified alginate and a natural corneal extracellular matrix was created by Zhao et al., and is known as an ion‐activated bioadhesive hydrogel (IonBAH) (**Figure** **6**). The liquid IonBAH is composed of corneal ECMs, functionalized alginates (APD), and transglutaminases (TGases). When applied directly to the area, it quickly closed the 6 mm corneal wound and restored the natural shape by employing a contact lens infused with calcium ions.^[^ ^99^ ^]^

Keratocyte behavior is influenced by variations in stiffness; however, static stiffness fails to adequately represent the dynamic characteristics of in vivo tissue. A hydrogel dynamicity was modulated by using a specially formulated mixture of alginate‐PEG (Alg‐PEG) and alginate‐norbornene (Alg‐Norb). Alg‐Norb functionalized with cell adhesive peptide (Alg‐Norb‐RGD) was also incorporated into the formulation to facilitate cell adhesion to an inert material. A photoinitiated norbornene‐norbornene dimerization process was used to modify the hydrogel's dynamic characteristics, resulting in relaxation periods that ranged from 30 s to 10 min. By employing this in vitro model, it is possible to optimize stress relaxation for corneal keratocytes to regulate tissue development.^[^ ^100^ ^]^

### 6.2 Chitosan

Chitosan (CS) is a carbohydrate polymer that has been chemically modified from chitin, which is present in several natural sources.^[^ ^101^ ^]^ It is widely used in medical and pharmaceutical fields.^[^ ^80^ ^]^ CS's antibacterial, biodegradable, biocompatible, non‐toxic, and mucoadhesive properties make it a popular biomaterial for tissue regeneration.^[^ ^102^ ^]^

**Figure 7:**
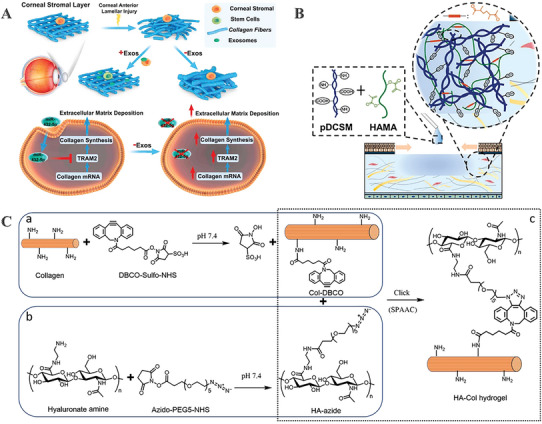
Development and evaluation of polysaccharide‐based corneal substitutes. A) The interplay of corneal stromal stem cells and exosomes during ECM remodeling following anterior lamellar damage. Adapted with permission.^[^ ^105^ ^]^ Copyright 2021, Elsevier Ltd. B) Schematic illustrations of the pDCSM‐G/HAMA hydrogel. Adapted with permission.^[^ ^57^ ^]^ Copyright 2022, The Authors. C) Hyaluronate‐collagen hydrogel crosslinked via SPAAC. Adapted with permission.^[^ ^110^ ^]^ Copyright 2020, Elsevier Ltd.

The presence of hydroxyl and amino groups in CS enables the development of self‐assembled nanoparticles that are utilized for targeted drug delivery by simple functionalization.^[^ ^103^ ^]^ Bakhshandeh et al., loaded human amniotic membrane extract (hAME) into chitosan‐dextran sulfate nanoparticles. These bioactive macromolecules enhanced the anti‐angiogenic properties of an artificial cornea by consistently emitting antiangiogenic factors.^[^ ^104^ ^]^ Tang et al., devised a unique approach that includes employing exosomes synthesized from induced pluripotent stem cell‐derived MSCs (iPSC‐MSCs) coupled with a thermosensitive hydrogel to prevent scar formation and expedite the healing process. Additionally, the miRNA (miR‐432‐5p) secreted by iPSC‐MSCs had a positive effect on the regrowth of corneal epithelial and stromal tissues. Exosome‐bound miR‐432‐5p decreased the accumulation of ECM following corneal damage by influencing the expression of its target gene, translocation‐associated membrane protein 2 (TRAM2) (**Figure** **7**A). iPSC‐MSC‐derived exosomes (iPSC‐MSC‐exos)‐based thermosensitive CS‐based hydrogels is a promising clinical treatment technology for a variety of corneal diseases.^[^ ^105^ ^]^

CS is an optimal chemical for the manufacture of nanoparticles due to its biocompatibility, antibacterial, antioxidant, and chelating characteristics.^[^ ^103^ ^]^ A transparent membrane made of CS, CS nanoparticles (CSNPs) and PCL has been developed to restore human ocular endothelial regeneration. The membrane improved the growth and attachment of human corneal endothelial cells (HCEnCs), leading to a dense single layer with no causing harm to the cells.^[^ ^106^ ^]^

### 6.3 Hyaluronic Acid

Hyaluronic acid (HA) is a straight‐chain polysaccharide that serves an essential part in the extracellular matrix, contributing to processes such as wound healing, cell movement, and cell communication.^[^ ^107^ ^]^ HA is an excellent material for tissue engineering applications because of its distinct physicochemical qualities including biodegradability, resorbability, biocompatibility, plasticity, and viscoelasticity. It is used in several forms such as sponges and hydrogels.^[^ ^108^ ^]^

In addition to its broad range of applications in regenerative medicine, HA frequently exhibits exceptional transparency, making it a highly suitable candidate for the restoration of corneal defects. Shen et al., devised a double‐network hydrogel that increased the adhesion of HA and its derivatives to the corneal epithelium and stromal cells. The hydrogel was composed of porcine‐decellularized corneal stroma matrix hydrogel (pDCSM‐G) and methacrylated hyaluronic acid (HAMA) (Figure 7B). A non‐competitive double crosslinking technique was employed, utilizing N‐cyclohexyl‐N’‐(2‐morpholinoethyl) carbodiimide metho‐p‐toluenesulfonate/N‐hydroxysuccinimide (CMC/NHS) and phenyl‐2,4,6‐trimethybenzoylphosphinate (LAP)‐induced photocrosslinking respectively. The bioactive components of pDCSM‐G promoted cell attachment, epithelial and stromal tissue regeneration, and related processes to prevent corneal fibrosis and scarring. The hybrid hydrogel was able to withstand the test of time because of the addition of HAMA.^[^ ^57^ ^]^ Another research used injectable biomaterials made of short peptides, glycosaminoglycans (including HAMA, chondroitin sulfate methacrylate (CSMA)), and GelMA that form a hydrogel when exposed to low‐energy blue light. To activate the material without damaging the cells, a minimal dosage of 8.5 mW cm^−^^2^ of pulsed‐blue light was enough. Two formulations of these light‐activated biomaterials were stable in the corneas of rats after injection, and neither version caused significant inflammation nor the development of new blood vessels.^[^ ^109^ ^]^

While photocrosslinking is a highly effective and adjustable process, it has some drawbacks when employed to create in situ gels on the cornea, such as the need for a photo‐initiator, the inefficiency of the process, and the presence of reactive byproducts. Chen et al., crosslinked hyaluronate and collagen (HA‐Col) using the bio‐orthogonal strain‐promoted azide‐alkyne cycloaddition (SPAAC) reaction at room temperature, without requiring photoinitiators, light, heat, or other chemical catalysts or initiators (Figure 7C). This hydrogel combined the cell‐adhesive characteristics of collagen with the growth‐promoting features of HA.^[^ ^110^ ^]^ Koivusalo et al., facilitated the transport of stem cells and promoted the regeneration of stromal tissues and corneal epithelium by incorporating dopamine components into hydrazone‐cross‐linked hyaluronic acid (HA‐DOPA) hydrogels. Dopamine components provided the tissue sticky property, enabled the attachment of cell‐adhesive proteins to the hydrogel surface, sustained the culture of human adipose stem cells (hASCs), and enhanced cell survival. Using this advanced delivery system, researchers demonstrated the feasibility of inserting two types of regenerative stem cells into the cornea using a model of pig corneal organ culture.^[^ ^111^ ^]^

### 6.4 Cellulose

Cellulose consists of glucose units connected by β‐1,4‐glycosidic linkages. Being biocompatible, biodegradable, water‐retention capacity, renewable, and adaptable makes cellulose a perfect biopolymer to use as a biomaterial.^[^ ^112^, ^113^ ^]^ Lee et al., introduced an innovative method involving cellulase treatment to produce human corneal limbal epithelial (HCLE) cell sheets from a surface coated with carboxymethyl cellulose (CMC)–dopamine (DA). In a rabbit model of limbal stem cell deficit, they showed that transplanting an HCLE cell sheet obtained from the CMC‐DA coating in combination with cellulase enzymatic harvesting is a safe and efficient therapeutic strategy. The method might provide an alternative method to regenerating corneal epithelium through transplantation for ocular surface diseases.^[^ ^114^ ^]^

Since nanotechnology has advanced, a new class of natural materials known as nanocellulose has gained interest from both academic and industrial research because of its exceptional mechanical qualities, high specific surface area, distinct nano morphology, and high crystallinity.^[^ ^115^ ^]^ Bacterial nanocellulose (BNC) is a newly developed biopolymer that exhibits considerable potential for skin tissue regeneration. As an alternative corneal bandage, BNC possesses a variety of desirable properties, including biocompatibility, flexibility, a high liquid‐holding capacity, and an abundance of functionalization opportunities.^[^ ^116^ ^]^ Anton–Sales et al., evaluated BNC as a carrier for human embryonic stem cell‐derived limbal stem cells (hESC‐LSC). Plasma activation was employed to optimize cell‐biomaterial interactions, which was subsequently followed by the application of collagen IV and laminin coatings onto the BNC substrates. The utilization of human extracellular matrix proteins to functionalize the surface of the BNC significantly enhanced the attachment and survival of hESC‐LSC, while maintaining the BNC's adaptable, durable, and partially transparent characteristics. The results demonstrated that hESC‐LSC maintained self‐renewal and stemness properties on BNC substrates for up to 21 days. These findings open the path for future studies employing hESC‐LSC/BNC constructions to treat severe ocular surface disorders.^[^ ^117^ ^]^

In a nutshell, polysaccharide‐based corneal implants can be designed to exhibit the characteristics of an optimal corneal construct, including transparency, mechanical strength, tissue adhesion, and biocompatibility. It is clear that the development of new manufacturing techniques to regulate the stable production of polysaccharide‐based corneal implants will be a promising clinical product in ophthalmology as tissue engineering and biomaterials science continue to advance.^[^ ^96^ ^]^

## 7 Synthetic Polymers

Polymers in the previous text are generated from things found in nature. Regenerative corneal surgery has also made use of synthetic polymers. Polyvinyl‐alcohol (PVA) is a transparent synthetic polymer known for its strong mechanical properties and compatibility with living tissues. To increase cell adhesion and bioactivity, PVA has been combined with natural chemicals to build corneal regeneration scaffolds. Chitosan blending decreased the light transmittance of PVA hydrogel while maintaining similar water absorption, degradation rate, and cytocompatibility to the native cornea.^[^ ^118^ ^]^ The mechanical characteristics, biocompatibility, and transparency of the membrane were enhanced by the layered arrangement of nanofibers with porous features.^[^ ^119^ ^]^

PCL is a hydrophobic polymer used in corneal endothelial stents, its hydrophobicity enables it to degrade slowly, which is a key component in managing the mechanical characteristics and degradation of chitosan nanoparticle/PCL composites.^[^ ^120^ ^]^ For the purpose of rebuilding corneal stromal tissue, a unique polyurethane‐urea (PUU) with biodegradable and tunable mechanical properties was created from poly (glycolide‐co‐ɛ‐caprolactone) macro‐diol. In order to regulate the release of vitamin C (VC) in the cell culture media, Zn‐Al layered double hydroxide (LDH) nanoparticles were created and then mixed with PUU (VC‐LDH).^[^ ^121^ ^]^ In order to avoid undesirable corneal vascularization, Zdraveva et al., studied electrospun PCL coated with anti‐vascular endothelial growth factor (anti‐VEGF). Surface‐adsorbed anti‐VEGF prevented vision loss and facilitated the healing of corneal tissue damage.^[^ ^122^ ^]^ Additionally, the inclusion of a decellularized corneal extracellular matrix in PCL has the potential to enhance the hydrophilic properties of scaffolds while maintaining Young's modulus unchanged.^[^ ^123^ ^]^

Poly (lactic‐co‐glycolic acid) (PLGA) a typical degradable biopolymer, has been studied extensively.^[^ ^124^ ^]^ Membranes containing 50:50 PLGA may be safely applied to rabbit corneas without creating any local or systemic toxicity, and they dissolve totally within 29 days.^[^ ^125^ ^]^ Naproxen sodium (NS)‐loaded PLGA scaffolds showed controlled drug release and biomimetic features, making them useful for corneal injury therapy in biomedical and controlled release applications.^[^ ^126^ ^]^

In contrast to natural polymers, the synthesis of synthetic polymers is highly reproducible, facilitating predictable chemical and physical properties, and it is uncomplicated to modify the characteristics of synthetic polymers to achieve desired results. Although synthetic polymers have shown encouraging results, additional in vivo research is required to confirm their safety and efficacy.

## 8 Conclusion and Outlooks

Researchers have taken a keen interest in corneal tissue engineering because of the potential benefits it could bring to the field of regenerative medicine. Ocular tissue engineering using biomaterials has shown promising results in clinical trials (**Table** **2**).

Collagen‐based materials exhibit the most potential as they closely resemble the natural makeup of the corneal stroma, although each material has its own distinct advantages and limits. Despite collagen's high biocompatibility, two significant issues have to be addressed: its rapid breakdown and weak mechanical characteristics. dECM materials can effectively preserve important components and original structures, creating an ideal microenvironment for healing, restoration, and regrowth. However, decellularization techniques need to be carefully tuned to reduce any potential toxicity. Gelatin may promote cell adhesion with little immunogenicity; it is biodegradable, water‐soluble, biocompatible, and non‐cytotoxic. Researchers are still grappling with the most pressing issue of all: how to synthesize bioactive hydrogels with adjustable stiffness and degradation characteristics. Although it has not been adequately studied, SF's high Young's modulus, flexibility, and thin film formation capabilities make it an appealing material for building ocular endothelial grafts, although its susceptibility to yellowing has not been properly addressed. Nanocarriers based on polysaccharides could improve medication delivery after ocular administration; these carriers could be polysaccharide‐matrix or polysaccharide‐coated, depending on their role.

To date, research has, to our knowledge, established a commercially and clinically viable cornea bioengineered from a sustainably sourced, cost‐effective, widely available, and FDA‐approved raw material that meets good manufacturing practices standards.^[^ ^25^ ^]^ The pursuit of regenerative therapies for ocular surface diseases is continuing.^[^ ^127^ ^]^ Data from patients who obtained corneal transplantation at the Transplant Unit of Athens General Hospital “Georgios Gennimatas” from 2020 to 2023 indicated that the average cost for AM surgeries was ≈1000 dollars, significantly lower than the average costs for Descemet's membrane endothelial keratoplasty and penetrating keratoplasty, which were ≈3000 dollars.^[^ ^128^ ^]^ We have reason to believe that in the future, with the large‐scale application of various biological materials in the field of corneal regeneration, the cost of transplantation surgery will have great space to decrease.

However, many obstacles must be addressed before achieving widespread clinical implementation of corneal tissue engineering. The primary characteristic is transparency. Yet there are few studies on the relationship between gel structure design and transparency, at the same time light transmittance data of the cornea after the implant wound has fully healed are rarely reported. Furthermore, it needs to be shaped and mechanically property‐preserving once implanted into the eye's physical environment, while enduring mechanical stretching and folding through tiny incisions, we may refer to this review article.^[^ ^129^ ^]^ Research should focus on eliminating barriers to producing a corneal substitute. Stem cells, 3D printing, and the development of biomaterials with highly tunable properties are among the future trends that will enable the precise control of the cornea's shape and structure. In another way, the biomaterials' surface can be modified to serve as substrates for the deposition of multifunctional coatings that inhibit antibacterial/scar formation or to function as inherent drug delivery devices. Advancements in biomaterials research, enhanced comprehension of corneal physiology, and the emergence of new technologies will result in superior corneal regeneration and transplantation outcomes.

**Table 1:** Advantages and limitations of common biomaterials for corneal regeneration.

Biomaterials	Advantages	Limitations	Refs.
Collagen	‐Similar to the corneal stroma's natural composition. ‐Modifiable optical, biocompatible, and physicochemical properties.	‐Induce immune responses, particularly when originating from various species. ‐Enzyme degradation, physical factors, and chemical agents all pose risks. ‐Production can be expensive, particularly when it involves large quantities and specific properties.	[23, 24, 25, 26, 27, 30, 31, 32, 33, 34, 36, 37, 38, 39, 40, 44, 45, 46, 47, 48, 49]
dECM Cornea	‐Exhibit biochemical identity with the original tissue, the intricate collagen structure in the corneal stroma can be preserved.	‐Require the removal of residual antigens after decellularization and remains of cytotoxic agents used in decellularization protocols.	[54, 58, 59, 60, 61, 64, 65, 67]
Gelatin	‐Biocompatible, hydrophilic, exhibiting minimal antigenicity and low immunogenicity. ‐Low cost.	‐Excessive biodegradability and inadequate mechanical properties.	[71, 72, 74, 75, 76, 77, 78, 79, 80, 81, 82, 85]
SF	‐Elevated Young's modulus, excellent flexibility, easy to form a film. ‐Biocompatible, biodegradable, with low immunogenicity.	‐Standardizing raw materials and their processing methods is challenging. ‐Difficult to stabilize for long‐term preservation.	[83, 87, 88, 89, 90, 91, 92, 93, 94]
Polysaccharide (Alginate, CS, HA, Cellulose)	‐Excellent biocompatibility, modifiable properties, easy processability, and high availability.	‐The final shape and quality of corneal implants may fluctuate due to batch‐to‐batch discrepancies. ‐Chitosan has the potential to be recognized as a foreign immunogen.	[57, 98, 99, 100, 104, 105, 106, 109, 110, 111, 114, 117]
Synthetic Polymers (PVA, PCL, PLGA)	‐Biological safety, tunable degradation characteristics, minimal immunogenicity. ‐The synthesis experiment has good repeatability.	‐Demands comprehensive processing and chemical modification. ‐PCL has poor hydrophobicity and transparency.	[118, 119, 121, 122, 123, 125, 126]

**Table 2:** Applications and Properties of Biomaterials for Corneal Regeneration.

Material types	Crosslinker/Combination Methods	Ocular Biological Models	Main Properties of the Materials	Major Results of in Vivo Experiments
Col‐I film^[^ ^23^ ^]^	Pure electro‐compacted	In vitro	Influence the phenotype of hCSCs in vitro to maintain a corneal keratocyte phenotype	–
Col‐I film^[^ ^24^ ^]^	Electro‐assembly, glutaraldehyde treatment	Rabbit lamellar keratectomy model	Enhanced transparency, and curvature can be customized	Support re‐epithelialization, and host tissue integration, enable the customization of complex 3D geometries and thickness
Col‐I membrane^[^ ^27^ ^]^	PRAs, EDC/NHS	Rabbit lamellar keratectomy model	Optimal light transmittance and water content, increased resistance to collagenase degradation	Facilitate effective corneal tissue regeneration avoiding inflammation or corneal neovascularization
Col‐I/dextran hydrogel^[^ ^30^ ^]^	Gd₂O₃ nanoparticle	In vitro	Down‐regulate VEGF‐A gene expression in endothelial cells, confirm compatibility with corneal tissue and lens crystallin proteins	–
Viscoll native collagen membrane^[^ ^32^ ^]^	Gelation, vitrification under constant pressure	Rabbit intrastromal keratoplasty model	Sufficient light transmittance and mechanical properties achieved without chemical crosslinkers	Double the central corneal thickness and strengthen corneal tissues while preserving transparency
Dual‐layered collagen vitrigel^[^ ^31^ ^]^	Beta cyclodextrin	Rabbit lamellar keratectomy model	High optical transparency and mechanical robustness	Complete re‐epithelialization and stromal integration with the recipient tissue
Col‐I/AuNPs/miR‐133b membrane^[^ ^33^ ^]^	Physical cross‐linking	Rabbit lamellar keratectomy model	Maintain MiR‐133b's integrity without affecting water content, light transmittance, and mechanical properties	Effectively prevent the formation of scars and promptly repair corneas
Col‐I/human MSCs hydrogel^[^ ^34^ ^]^	Multi‐arm PEG/NHS	Rabbit corneal alkali burn injury model	Enable the containment of functional MSCs	Recover some transparency, less stromal vimentin was detected
RHCI hydrogel^[^ ^36^ ^]^	EDC/NHS	Minipig anterior keratoplasty model	Exhibit high yields, transparency, mechanical stability, and biocompatibility	Facilitate the regeneration of corneal epithelium, stroma, and nerves
RHCMA in situ hydrogel^[^ ^26^ ^]^	LAP/UV irradiation	SD rat intrastromal keratoplasty model	Physiochemical properties similar to native cornea, with aligned microgrooves	Speed up the healing process and eventually allow damaged stromal tissue to grow back
BPCDX hydrogel^[^ ^25^ ^]^	EDCM, UV irradiation	Minipig femtosecond laser‐enabled intrastromal keratoplasty model	Preserve transparency, enzymatic resistance, water content, and mechanical properties over 24 months of aging	Enhancements in corneal thickness, maximum keratometry, and visual acuity 20 advanced keratoconus subjects
CLP‐PEG‐fibrinogen liquid hydrogel filler^[^ ^37^ ^]^	DMTMM	Rabbit lamellar keratectomy model	Completely gelatinized within 5 min at body temperature	Allow corneal epithelium, stroma, and nerve regeneration, decreased xenogeneic material allergy and immunological rejection risks
CLP‐PEG‐MPC hydrogel^[^ ^38^ ^]^	DMTMM	Minipig corneal alkali burn injury/anterior lamellar keratoplasty model	Exceedingly clear while filtering up to 60% of potentially harmful UV‐A rays, properly robust	Inflammation suppression with entirely synthesized collagen analog
CLP‐PEG liquid hydrogel filler^[^ ^39^ ^]^	DMTMM	Feline stepwise surgical corneal ablation model	–	Allow steady corneal shape and transparency repair in 12 months with less irritation and no neovascularization
FSCM/CECs membrane^[^ ^40^ ^]^	1,4‐butanediol diglycidyl ether	Rabbit corneal anterior chamber implantation model	Superior transparency, sufficient water content, and great biocompatibility	Provide an ideal endothelial keratoplasty transplantation CEC carrier
Deep frozen (cryopreserved) AM membrane^[^ ^44^ ^]^	Using polyester net	In vitro	Beneficial carrier for human keratinocytes and corneal limbal stem cells	–
AM‐HEP membrane^[^ ^45^ ^]^	Surface graft heparin	Mouse corneal alkali burn model	Heparin maintains stability for three weeks at 37 °C, and the sustained release of EGF lasts for almost 14 days	Substantially enhance corneal epithelial cell proliferation and motility, reduce inflammation, and improve transparency
AM/PDMS membrane^[^ ^46^ ^]^	Electrospinning	Rabbit corneal epithelial injury model	Enhancement of biomechanical properties (mechanical strength, degradation, and transparency)	Corneal transparency increased with enhanced epithelial delaminated cell morphology
PCL nanofiber‐dAM^[^ ^47^ ^]^	Electrospinning	Rabbit alkali burn‐induced LSCD model	Preserve the pro‐regenerative and immunomodulatory characteristics of dAM, enhance LSC survival, retention, and organization	Facilitate re‐epithelialization of the defect region, and decrease inflammation and neovascularization
mcdAM scaffold^[^ ^48^ ^]^	Corneal crosslinking and ECM‐coated methods	Cat/monkey Descemet's membrane endothelial keratoplasty model	Effectively improve mechanical properties and cell adhesion	Maintain corneal transparency, standard morphology, and histological integrity of the regenerated corneal endothelium
COMatrix hydrogel^[^ ^60^ ^]^	Incubation at 37 °C	Murine corneal epithelial debridement model	Increase HCEC attachment and proliferation	Speed corneal epithelial wound closure, enhance the expression of the proliferation marker Ki‐67 in injured corneal epithelium
LC‐COMatrix in situ hydrogel^[^ ^54^ ^]^	Eosin Y/TEOA/VC/green LEDs irradiation	Rabbit corneal perforation model/rabbit stromal‐defect model	Promote biomechanical strength, stability, and adhesiveness	Seal extensive corneal holes, and restore partial‐corneal stromal defects
dCMH in situ hydrogel^[^ ^59^ ^]^	Incubation at 37 °C	In vitro	Maintain keratocytes that can regenerate corneal defects without scarring	–
APCS‐Asp scaffold^[^ ^61^ ^]^	EDC/NHS	Rabbit lamellar keratectomy model	Suggest appropriate cell sources for in vitro reconstruction of the ATELC epithelial layer	Restore corneal shape and optical performance within a week
Co‐dECM sealant^[^ ^58^ ^]^	Ru/SPS, visible blue light irradiation	Rabbit lamellar keratectomy model	Contain biofactors and adherable proteins	Successfully regenerated tissues possess matrices close to normal tissue with minimal scar formation
Fibrin–dECM hydrogel^[^ ^64^ ^]^	Incubation at 37 °C	Rabbit anterior lamellar keratectomy model	Optimal particle size distribution (≤10 µm) of dECM microparticles, maintain the majority of corneal ECM constituents present in native tissue	Accelerate corneal re‐epithelialization within 14 days and stimulate stromal tissue restoration
Co‐dECM hydrogel^[^ ^65^ ^]^	3D printing	Rabbit corneal pocket model	Comparable quantitative measurements of collagen and GAGs relative to those of the native cornea	An in vivo safety comparable to clinical‐grade collagen, assists in sustaining the particular properties of keratocytes
GelNB eyedrops^[^ ^71^ ^]^	EDC/NHS, UV irradiation	Rabbit partial‐thickness‐keratectomy model	Establish a thin covering on the corneal surface, avoid fast clearance	Promote cell migration to speed corneal healing and regeneration
Gelatin/lactose film, gelatin/citric acid film^[^ ^72^ ^]^	Self‐crosslinking	In vitro	Good optical and biological properties	–
GelMA in situ hydrogel^[^ ^74^ ^]^	LAP/near‐UV blue light irradiation	In vitro	Assess GelMA hydrogels' physicochemical and biological qualities at various concentrations	–
Corneal stromal cells loaded with GelMA hydrogel^[^ ^75^ ^]^	Eosin Y/TEA/NVP/UV light irradiation, SLA 3D bioprinting	In vitro	An exceptional substrate for the adhesion and proliferation of corneal stromal cells	–
GelMA hydrogel, HAGM hydrogel^[^ ^76^ ^]^	LAP/DLP‐based bioprinting	In vitro	To support active and quiescent statuary, develop a bioprinted dual ECM “Yin‐Yang” model that utilizes LSCs	–
GelCORE bioadhesive hydrogels^[^ ^77^ ^]^	TEAVC/Eosin Y disodium salt/visible light irradiation	Rabbit lamellar keratectomy model	High transparency and tissue adhesion, fine‐tuning of physical properties, be able to in situ photopolymerization	Improved stromal regeneration and re‐epithelialization, increased tissue adhesion, and closure of corneal abnormalities
GelMA/PECL 3D fiber‐reinforced hydrogel^[^ ^78^ ^]^	LAP/UV irradiation	Rat corneal pocket model	Modify the fiber spacing to construct the stromal architecture with properties closely resembling those of the original cornea	Facilitate keratocyte phenotypic maintenance and corneal stroma renewal
Gelatin‐PCL scaffold^[^ ^80^ ^]^	Electrospinning	In vitro	HCSC grown on scaffolds become therapeutically quiescent	–
GelMA/F127DA/AF127/Col‐I in situ hydrogel^[^ ^79^ ^]^	riboflavin‐5‐phosphate/UV irradiation	Rabbit lamellar keratectomy model	High transparency, durable, bio‐adhesive, able to in situ photopolymerization	Replace profound corneal stromal deficiencies, bio‐integrate into corneal tissue within 4 weeks
Gel‐MA/OHA hydrogel^[^ ^81^ ^]^	Irgacure 2959/UV irradiation	Rabbit lamellar keratectomy model	Physicochemical properties can be controlled, and employed as a drug carrier to provide regulated release	Efficiently repair the defect, speed epithelial formation, and stimulate the regeneration of a corneal analog
Gelatin microcarriers with OHA^[^ ^82^ ^]^	EDC/NHS	Rabbit corneal alkali burn injury model	Enhance the microcarrier surface with a smooth topography and great stiffness	Significantly eliminate corneal inflammation and restore disorganized collagenous stromal structure after 4 weeks
SNF/GelMA‐SA film^[^ ^85^ ^]^	Micro‐molding approach	In vitro	For corneal regeneration, make a double‐layer film with micro‐patterned patterns that adhere firmly to tissue and provide AA with a continuous release	–
SF (derived from PR/AA BM) film^[^ ^87^ ^]^	Casting	In vitro	PR and AA types provide enough support for the growth and operation of CE cells	–
G/SF film^[^ ^88^ ^]^	Casting	In vitro	Suitable carrier for CEnCs	–
Tropoelastin‐silk hybrid film^[^ ^83^ ^]^	Casting	In vitro	Transparent, glucose‐permeable, mechanically compliant, supports corneal epithelial and endothelial cell development and function	–
Silk film^[^ ^89^ ^]^	Casting	In vitro	For ocular surface repair, silk film rigidity regulates cell activity	–
SF/G film, SF/G scaffold^[^ ^90^ ^]^	Casting, dehydrothermal technique	In vitro	Improve cell adhesion, viability, proliferation, and keratocyte differentiation	–
PCL‐SF scaffold^[^ ^91^ ^]^	Electrospinning	In vitro	Excellent physical, chemical, and biological qualities for corneal stromal regeneration	–
SF‐based scaffold containing GDNF^[^ ^92^ ^]^	Casting	Rat epithelial‐stromal corneal damage model	The cultured corneal stromal cells show a high level of vitality and proliferation activity	Stimulate epithelialization, cell proliferation, stromal nerve plexus development, and anti‐apoptotic action
Gentamicin‐loaded SilkMA in situ hydrogel^[^ ^93^ ^]^	LAP/UV irradiation	In vitro	Biodegradable, biocompatible, antibacterial, and cornea‐like optical and mechanical qualities	–
PA/SF hydrogel^[^ ^94^ ^]^	N, N‐MBAAm	In vitro	Excellent cell viability, optical properties, and phenotypic	–
CMCTS/SAD in situ hydrogel^[^ ^98^ ^]^	Self‐crosslinking	Rabbit corneal alkali burn injury model	Biocompatible and biodegradable, trigger just a minimal inflammatory reaction immediately post‐injection	Transplant‐encapsulated LSCs to alkali‐burned corneas accelerate tissue regeneration and wound healing
ECM/peptide‐modified alginate^[^ ^99^ ^]^	APD, TGases	Rabbit lamellar keratectomy model	Preferred transparency and biocompatibility, adjustable mechanics, and strong adhesion	Restore transparency, normal curvature, corneal epithelium, stroma, and nerves, and quickly close the 6 mm corneal defect
Alg‐Norb/Alg‐Norb‐PEG/Alg‐Norb‐RGD hydrogel^[^ ^100^ ^]^	Calcium, LAP/UV irradiation	In vitro	Optimize stress relaxation for different cell types to govern tissue development	–
hAME‐loaded chitosan‐dextran nanoparticle^[^ ^104^ ^]^	Polyelectrolyte complexion method	In vitro	Increase artificial cornea anti‐angiogenicity	–
iPSC‐MSC‐exos‐based CS hydrogel^[^ ^105^ ^]^	Incubation at 37 °C	Rat anterior lamellar keratectomy model	Exosomes contain miRNA, which can be directly transported into target cells	Improve corneal epithelium and stroma regeneration, prevent ECM deposition probably by directly repressing miR‐432‐5p's target gene TRAM2
CSNP/PCL membrane^[^ ^106^ ^]^	Sodium three‐polyphosphate	In vitro	Transparent and biocompatible for corneal endothelial regeneration	–
pDCSM/HAMA in situ hydrogel^[^ ^57^ ^]^	CMC/NHS, LAP/UV irradiation	Rabbit lamellar keratectomy model	Attain suture‐free healing, exhibit cornea‐matching transparency, possess a low swelling ratio, demonstrate gradual degradation, and enhance mechanical properties	Quickly and painlessly heal corneal defects without sutures, promote long‐term tissue regeneration for complete tissue recovery
8‐Arms‐PEG acrylate/CSMA/ HAMA/GelMA/ (short peptides) in situ hydrogel^[^ ^109^ ^]^	(APS/TEMED)/riboflavin/blue light irradiation	Rat corneal pocket model	Low‐energy blue light irradiation triggers polymerization and reduces cytotoxicity	Two formulations are stable in situ without causing inflammation or neovascularization
HA‐Col in situ hydrogel^[^ ^110^ ^]^	SPAAC click reaction	Rabbit lamellar keratectomy model	Highly transparent, no trigger or initiator needed	Great cytocompatibility and epithelialization support
HA‐DOPA hydrogel^[^ ^111^ ^]^	Hydrazone crosslinking chemistry	In vitro	Enhance cell‐adhesive proteins bind to hydrogels, enhance hASC culture	–
HCLE cell sheets cultured on CMC‐DA combined with cellulase^[^ ^114^ ^]^	EDC/NHS	Rabbit limbal stem cell deficiency model	HCLE cell sheets are generated with optimal shape and transparency	Raise the neovascularization and corneal opacity ratings
BNC hydrogel^[^ ^117^ ^]^	Produced from Komagataeibacter xylinus	In vitro	Maintain the hESC‐LSC progenitor phenotype and enhance daughter cell production	–
PVA/CS composite^[^ ^118^ ^]^	3D printing	In vitro	Efficient biocompatibility, light transmittance, and mechanical properties	–
3D keratin/PVA NFs membrane^[^ ^119^ ^]^	Electrospinning	Rabbit lamellar keratectomy model	Better transparency, biocompatibility, and mechanical qualities provide ECM‐like 3D fiber networks	Can be considered as an alternative material to the horse AM
PUU‐VC‐LDH nanofiber film^[^ ^121^ ^]^	Electrospinning	In vitro	Suitable mechanical qualities, promote adhesion and proliferation, increase vimentin secretion	–
PCL/anti‐VEGF scaffold^[^ ^122^ ^]^	Electrospinning	In vitro	Enhanced stiffness, biodegradation rate, and the adhesion of cultured limbal stem cells	–
PCL/ECM scaffold^[^ ^123^ ^]^	Electrospinning	In vitro	Promote increased cell migration with radial and perpendicularly oriented fibers	–
PLGA membrane^[^ ^125^ ^]^	Electrospinning	Rabbit limbal stem cell deficiency model	–	Safely administered to rabbit corneas without causing any local or systemic harm, disintegrate entirely within 29 days
NS‐incorporated PLGA scaffold^[^ ^126^ ^]^	Emulsion freeze‐drying method	In vitro	Biomimicry and regulated medication release	–

## Conflict of Interest

The authors declare no conflict of interest.

## Author Contributions

Z.W. proposed the topic of the review. Y.L. investigated the literature and prepared the manuscript. Z.W. revised the manuscript.
